# Flucytosine for treatment of *Candida albicans* in H1N1-positive patient

**DOI:** 10.4103/0253-7613.70398

**Published:** 2010-10

**Authors:** Tanvir Samra, Amlendu Yadav, Neerja Banerjee, Mridula Pawar, Desh Deepak

**Affiliations:** Department of Anaesthesia and Intensive Care, Dr Ram Manohar Lohia Hospital, Baba Kharak Singh Marg, Connaught Place, New Delhi - 110 001, India

**Keywords:** Flucytosine, empyema thoracis, *Candida albicans*, H1N1 infection

## Abstract

A 17-year-old H1N1-positive patient was successfully extubated after 25 days of ventilatory support for treatment of viral pneumonia which was complicated by empyema thoracis due to *Candida albicans*. Hematogenous spread was suspected as simultaneous culture of ascitic fluid identified *Candida* species sensitive to flucytosine but resistant to amphotericin B and azole group of antifungals. Monotherapy with flucytosine led to clinical and radiological improvement.

## Introduction

Viral pneumonia was considered mild till the emergence of H1N1 pandemic. Its propensity to infect cells deeper in the lungs and cause severe lung injury necessitate the need for mechanical ventilation. Prolonged stay in intensive care unit (ICU) increases the chance of acquiring nosocomial infections. We report empyema thoracis due to *Candida albicans* in a H1N1-positive patient treated with flucytosine.

## Case Report

A 17-year-old schoolboy presented to the emergency department with fever, productive cough, and breathlessness since 4 days. He was conscious, oriented, and hemodynamically stable with Sp O_2_ of 85% in room air which increased to 92% with facemask. H1N1 infection was suspected and the patient was admitted in isolation ward. Nasal and throat swabs were sent for real-time reverse transcription--polymerase chain reaction (RT-PCR) which tested positive for the virus. Over next 24 h there was clinical deterioration and fall in PaO_2_ to 49 mmHg. Chest X-ray showed right-sided consolidation involving all four zones. Trachea was intubated and patient shifted to ICU for mechanical ventilation.

Reports of hemogram, liver function test, renal function test, and serum electrolytes were normal. Blood, endotracheal aspirate, and urine were sent for bacterial culture on alternate days. Antibiotics were prescribed based on the report of culture sensitivity [Table 1]. Oseltamivir was continued for a total duration of 20 days at a higher dose of 150 mg BD. Though there was steady improvement in lung oxygenation for next 10 days, the patient continued to have pyrexia (104°F) and a rising total leucocyte count (upto 30,000/mm^3^).

**Table 1:** Report of culture sensitivity and antibiotics administered

*Specimen*	*Organism*	*Sensitivity*	*Antibiotics*	*Duration (in days)*
Endotracheal aspirate	*Acinetobacter*	Piperacillin/Tazobactam	4.5 g TDS	5 (D1-D5)
Endotracheal aspirate	*Pseudomonas*	Levofloxacin	500 mg OD	7(D6-D12)
Endotracheal aspirate	*S. aureus*	Tetracycline	250 mg QID	7(D13-D19)
Endotracheal aspirate	*S. aureus*	Meropenem	1 g TDS	D20-D24
Pleural and ascitic fluid	*Candida albicans*	Flucytosine	50 mg/kg/day divided into four doses	D14-D18

*Anaerobic cover was given with clindamycin

Further attempts to decrease ventilatory support were hindered by chest X-ray finding suggestive of right-sided pleural effusion. Ultrasound of the chest done on day 12 localized an appropriate site for drainage and also reported moderate amount of free fluid in the abdomen. Right-sided chest drain was inserted aseptically and both pleural and ascitic fluid were sent for bacterial and fungal culture which identified *Candida albicans (C. albicans)* sensitive to flucytosine but resistant to both amphotericin B and azole group of antifungals. Simultaneous specimens of blood were also sent for bacterial and fungal cultures which were reported as negative. Flucytosine in daily dose of 2.5 g in four divided doses was administered for a duration of 5 days. No adverse effects were noted. The repeat sample of pleural fluid sent for fungal culture after 4 days tested negative and chest drain was removed on sixth day. The patient became afebrile, leucocyte count decreased (15,000/mm^3^), and weaning from mechanical ventilation was initiated. There was radiological improvement and the patient was successfully extubated after 25 days of ventilatory support. He was shifted toward after 4 days of oxygen therapy with ventimask in the ICU and from there discharged home on room air after 35 days of hospitalization.

## Discussion

Fungal empyema thoracis is associated with mortality as high as 73%, and thus necessitates early administration of antifungal agents and pleural drainage.[1] Guidelines published by Infectious Diseases Society of America recommend either fluconazole or an echinocandin (Caspofungin) for treatment of candidemia in non-neutropenic patients.[2] The duration of therapy is 2 weeks after documented clearance of *Candida* from the bloodstream or resolution of symptoms attributable to candidemia. Amphotericin B deoxycholate is no longer used as first line therapy as it causes infusion toxicity and nephrotoxicity. Also with increasing use of antifungals, resistance to amphotericin B and azoles have developed in some strains of *C. albicans*. New antifungals like triazole compounds, caspofungin, combination therapy of various antifungals, and immune therapy are alternatives in such cases.

Flucytosine is an antifungal drug which is administered in combination with amphotericin B for patients with invasive disease. Monotherapy with flucytosine is effective in treating infections caused by *Candida* species[3] but is avoided as it leads to development of resistance during treatment.[4] Candida species isolated in our patient was sensitive to flucytosine but resistant to amphotericin B and azole group of antifungals. Due to high cost caspofungin was not administered and we decided to initiate monotherapy with flucytosine and monitor the patient for clinical improvement and microbiological clearance. We limited duration of therapy to 5 days to avoid development of resistance as subsequent samples of pleural fluid had already tested negative for fungus. Experimental studies indicate variable duration of treatment with flucytosine based on the severity of disease and clinical condition of patient. The therapeutic efficacy of flucytosine monotherapy has been established in a mouse model of acute invasive aspergillosis with survival rates as high as 90%. Duration of therapy was 7 days and the efficacy was found to be dependent primarily on the total daily dose administered.[5]

Good response was seen in this case probably because the patient was young with no other co-morbidity. The risk factors for candidiasis present in our patient were prolonged stay in ICU, central venous catheter, and prolonged use of antibiotics. The route of infection in pulmonary candidiasis is either by hematogenous dissemination or as a result of aspiration of oropharyngeal and gastric contents.[6] The presence of *C. albicans* in both pleural and ascitic fluid in our patient favours haematogenous spread in spite of negative blood culture reports. It is to be noted that some patients with deep organ infection have negative blood culture reports but this does not rule out systemic candidiasis.

There are very few case reports of fungal infections in patients with H1N1 pneumonia. One such report describes two male patients with invasive aspergillosis after infection with H1N1 pandemic.[7] They were admitted with acute respiratory distress syndrome (ARDS) and mechanically ventilated for 70 and 21 days before their death. There was lymphopenia on admission and subsequent development of renal failure, but no pre-existing co-morbidity. High-dose methylprednisolone (1 mg/kg/ day) and broad-spectrum antimicrobial drugs predisposed these patients to invasive aspergillosis. Antiviral treatment was not started as diagnosis was made > 48 h after the onset of symptoms. Antifungal used for initial therapy in both patients was intravenous voriconazole (6 mg/kg every 12 h, followed by 4 mg/kg every 12 h) for 24 and 21 days to which none of the patients responded. The one with longer duration of hospital stay was also given micafungin (100 mg/d) and amphotericin B lipid complex for 2 weeks.

Cell-mediated defects, leukopenia, structural defects in pulmonary parenchyma due to ARDS, and immunodysregulation caused by H1N1 predispose patients for fungal diseases. Such patients are also at risk of secondary bacterial infections with *Streptococcus pneumoniae, Haemophilus influenzae* and *Staphylococcus aureus* and ‘broad-brush’ approach to antibiotic prescribing advocates use of ‘broad-spectrum cephalosporins or quinolones for inpatients with H1N1 pneumonia.[8] This increases the risk of *Clostridium difficile*-associated diarrhoea (CDAD), methicillin-resistant *S. aureus* (MRSA) and superimposed fungal infections. High index of suspicion helps in early detection and efficient management of these life-threatening nosocomial infections.
